# Sociodemographic and health care factors in determining immunization defaulters among preschool children in Petaling District, Selangor: a cross-sectional study in Malaysia

**DOI:** 10.1186/s12889-019-7561-z

**Published:** 2019-09-18

**Authors:** Damyanthy Krishna, Nor Afiah Mohd Zulkefli, Salmiah Md Said, Aidalina Mahmud

**Affiliations:** Department of Community Health, Faculty of Medicine and Health Sciences, University Putra Malaysia, 43400 Serdang, Selangor Malaysia

**Keywords:** Immunization, Defaulters, Prevalence, Malaysia

## Abstract

**Background:**

Immunization is an effective public health intervention to reduce morbidity and mortality among children and it will become more effective if the child can receive the full course of recommended immunization doses. The objective of this study was to determine the prevalence of childhood immunization defaulters and its associated factors among children below 5 years attending registered child care centers in Petaling District, Selangor.

**Methods:**

This was a cross-sectional survey among mothers with children below 5 years from 60 registered child care centers in District of Petaling, Selangor. Data was collected by a self-administered questionnaire from a total of 1015 mothers. Simple Logistic Regression, Chi-square or Fisher’s exact test were performed to determine the association between individual categorical variables and childhood immunization defaulters. Multivariate logistic regression was used to determine the predictors of childhood immunization defaulters.

**Results:**

The study showed that the prevalence rate for defaulting immunization was 20.7%. After adjusting all confounders, six statistically significant predictors of childhood immunization defaulters were determined. They were non-Muslims (aOR = 1.669, 95% CI = 1.173, 2.377, *p* = 0.004), mothers with diploma and below educational background (aOR = 2.296, 95% CI = 1.460, 3.610, *p* < 0.0001), multiple children of 5 and above in a family (aOR = 2.656, 95% CI = 1.004, 7.029, *p* = 0.040), mothers with younger children aged 2 years and below (aOR = 1.700, 95% CI = 1.163, 2.486, *p* = 0.006), long travelling time of more than 30 min to the immunization health facility (aOR = 2.303, 95% CI = 1.474, 3.599, *p* < 0.0001) and had delayed at least one of the immunization schedule (aOR = 2.747, 95% CI = 1.918, 3.933, *p* < 0.0001).

**Conclusion:**

This study highlights the need of implementation of intervention programs should be intensified to improve the childhood immunization status, focusing on the Non-Muslim community, mothers with low educational level, mothers with multiple children and mothers with children aged 2 years and below. In light of the growing problem of immunization defaulters in Malaysian children, identifying mothers at risk of not completing their children immunization schedule and educating them is an important strategy to recurrent outbreaks of infectious disease in the country.

**Electronic supplementary material:**

The online version of this article (10.1186/s12889-019-7561-z) contains supplementary material, which is available to authorized users.

## Background

According to the World Health Organization (2013), immunization is a process whereby a person is made immune or resistant to an infectious disease, typically with the administration of a vaccine (Immunization is a strategy of WHO to reduce the mortality rate of under-five year old children against eight vaccine-preventable diseases; tuberculosis, diphtheria, whooping cough (pertussis), tetanus, hepatitis B, polio, measles and respiratory diseases caused by *Haemophilus influenza.* Thus, to ensure immunization coverage sustains throughout the world, WHO launched the Expanded Program on Immunization (EPI) in 1974 and has significantly reduced the incidence of vaccine preventable diseases while preventing more than 2 million child deaths each year [1]. Thus, worldwide studies show that the cost to treat a vaccine preventable disease is 30 times more than the cost of the vaccine itself [2].

Despite this, the implementation of immunization programs varies greatly across different communities and there are at least 30 million children worldwide who are not routinely immunized [3]. In 2016, World Health Organization (WHO) reported that only 86% of children worldwide received three doses of diphtheria-tetanus-pertussis (DTP3) vaccine while an estimated 19.4 million children missed out these basic vaccines [1]. On the other hand, vaccine preventable diseases namely measles, whooping cough (pertussis) *Haemophilus influenza* type B (Hib) and tetanus remain a major disease burden among children in developing countries. The World Health Organization (WHO) and the United Nations Children’s Funds (UNICEF) estimate that at least 1.5 million children under 5 years of age continue to die each year from vaccine-preventable diseases due to inadequate immunization coverage such as defaulted immunization, delayed immunization, incomplete or non-immunization, mainly in Sub-Sahara Africa (SSA) and South-East Asia [3].

Malaysia being one of the developing nations in Southeast Asia, has also adopted the WHO’s Expanded Programme of Immunization (EPI) in so as to reduce the burden from vaccine preventable diseases. The national immunization program based on the Ministry of Health’s immunization schedule, stipulates that children should receive eight basic primary immunization and be completely immunized with the following vaccines by 12 months of age: one dose of Bacillus CalmetteeGuerin (BCG) and hepatitis B (HepB) vaccines at birth (or within 24 h of birth); two more doses of HepB vaccine at 1 and 6 months of age; three doses of diphtheria, tetanus and pertussis with *Haemophilus influenzae* type b (Hib) and inactivated poliovirus (IPV) (DTaPHib//IPV) at 2, 3 and 5 months of age; and one dose of mumps, measles, and rubella (MMR) vaccine at 12 months of age [4].

In the past decade, Malaysia has achieved high immunization coverage of more than 90% reported among children [4]. Despite its overwhelming success, childhood immunization is becoming a growing concern and huge challenges still persist in the country where outbreaks of vaccine-preventable diseases such as diphtheria and measles still occur sporadically [4, 5]. Nonetheless, an increasing trend in vaccine hesitancy is being tracked by the Health Ministry of Malaysia (MOH) whereby a considerable number of parents with young children are refusing and defaulting immunization schedule, from 470 cases in 2013 to 1292 cases in 2014, based on data collected from government clinics and hospitals [6]. This underlines the particular need for continued monitoring of immunization program performance to detect potential problems and identify appropriate solutions.

Adapting from previous studies, factors associated with childhood immunization defaulters are maternal socio-demographic characteristics including maternal age, ethnicity, religion, marital status, educational level, working status, family composition and number of children, while child factors consist of child’s age, gender and birth order [7, 8]. Some factors related to the healthcare services that have been correlated with immunization include place of immunization, distance to immunization health facility, travelling time, waiting time to receive immunization and delayed immunization schedule may determine the health seeking behaviors of mother to default or immunize their children [9, 10]. However, these factors may vary across and within cultures, and differs from one geographic and social setting to another [10].

A Malaysian study in Sabah conducted in government maternal child health clinics reported a defaulter rate of 16.8%. The study noted that working mothers with bigger family size were significant factors associated with defaulters [11]. However, there was no study in Malaysia that investigated the relationship between immunization defaulters and the socio-demographic and healthcare factors among young children attending child care centers. Thus, this study aims to determine the prevalence and the predictors of childhood immunization defaulters among children below 5 years attending child care centers in Petaling District, Selangor. The findings from this study will add new knowledge in the respective field and will provide useful information for the government or policy makers in terms of planning an intervention or a campaign targeting mothers who are at risk of defaulting immunization by focusing on the significant predictors of this study.

## Materials and methods

### Study location, study design, and study duration

This study was a cross sectional study conducted among mothers of children aged 5 years and below attending child care centers in Petaling District, Selangor, Malaysia. Malaysia comprises of 13 large states and 3 different federal territories. Its area is about 330,803 km^2^. The Malaysian land is divided into two parts, namely Malaysian Borneo and Peninsular Malaysia, between which the South China Sea flows. The population of Malaysia was estimated in 2018 to be around 32.04 million [12]. Petaling is one of the districts located in the state of Selangor in Peninsular Malaysia. Petaling District covers an area of 484.32 km^2^ and has an estimated population of 1,782,375 in 2016 [13]. It has the highest population density and has been recognized as one of the most developed districts in Malaysia. This study was carried out over a period of 5 months, from July to November 2016.

Preschool education provided in child care centers known as Taska in Malaysia, caters to the education needs of children aged below 5 years [14]. The list of all child care centers registered under the Department of Social Welfare Malaysia located in Petaling District was used as a sampling frame. Particularly, there are 160 registered child care centers in Petaling District [15]. Child care centers were selected because they can yield the latest immunization data of young children below 5 years and capture the population of mothers utilizing the immunization services from both private and government health facilities. The age-range of 5 years and below was selected because most recommended immunizations would have been received by then. The study population was mothers with children aged 5 years and below who are attending the registered child care centers in Petaling District. Mothers were asked to serve as respondents in order to standardize data collection and because mothers are the primary caregivers of these children, thus they play an important role in the children’s health and well-being [2].

### Sample size

The sample size was estimated by using a two-proportion formula for hypothesis testing. It was determined using the Lemeshow, Hosmer, Klar, and Lwanga, (1990) formula with requirement for significance level (α) of 0.05 and 90% power, with the assumption of 5% margin of error, 95% confidence level [16]. A total of 996 respondents were required after adjustment for comparison of two groups, design effect of 2, estimated 10% non-response rate and 10% ineligibility.

### Sampling method

Cluster sampling technique was employed for the selection of child care centers in Petaling District. Each selected child care centers were considered as cluster and was randomly selected using the two-digit table of random numbers. A total of 60 child care centers were approached to participate in this study and the principals gave permission to conduct this study in their institutions. All the mothers of children aged five and below from the selected child care centers were invited to participate in this study. Informed consent was obtained for participation in the study. Inclusion criteria were mothers with children aged 5 years and below attending registered child care centers during the study period. While, those mothers who refused to participate were mothers that were non-Malaysian citizens and children without their immunization record were excluded from this study.

### Definition of study variables

The dependent variable in this study was immunization defaulters. Defaulters were defined as children who had defaulted on one or more doses of immunization from birth to their respective age group while those children who received all doses of immunization for their age were defined as immunization non-defaulters [2].

The independent variables were socio-demographic characteristics (maternal age, religion, ethnicity, educational status, marital status, working status, number of children), child factors (age, gender and birth order) and healthcare factors (place of immunization, distance to the preferred immunization facility, travelling time, waiting time to obtain immunization and delayed immunization schedule). These variables were chosen based upon prior studies illustrating their association with immunization defaulters.

### Study instruments and data collections

For the purpose of data collection, a 17-item pre-tested questionnaire was developed as the instrument for this study (Additional file 1). The questionnaire consisted of three sections that covered maternal, child, immunization history and health service related factors. The questionnaire was adapted and modified from the Expanded Program of Immunization (EPI) surveys [17] and existing literature [11, 18]. The immunization status of the children was assessed as per the recommended national immunization schedule used during the time of study. Mothers were asked to use their child’s immunization records to indicate the recommended vaccines and doses their children had received, focusing on the eight-basic primary immunization recommended by the Ministry of Health Malaysia; BCG, Hepatitis Dose 1, 2 and 3, Diphtheria-Tetanus-Pertussis-*Haemophilus Influenza* (Hib) Dose 1, 2 and 3, and Measles-Mumps-Rubella. The information given by the respondents were then further counterchecked with the Ministry of Health Malaysia Child’s Health Booklet to reduce the likelihood of inaccurate reporting and avoid recall bias.

A pilot study was carried out among 100 mothers of children below 5 years who were randomly selected from four registered child care centers in Gombak District which was not selected in the main study. The questionnaires were pre-tested for face validity, content validity and internal consistency. The content validity was done by professionals from the Faculty of Medicine and Health Sciences, University Putra Malaysia. The professionals were three experts, who are specialists in family medicine, community health and family health fields. The results of the reliability test of the questionnaire showed that the internal consistency was acceptable with Cronbach’s alpha ranged from 0.712 to 0.891. The questionnaire was bilingual, in English and Bahasa Malaysia language (national language of Malaysia). The questionnaire was translated from English to Malay by a professional language translator. The Malay version was used for the better understanding of the respondents.

### Data analysis

The data collected were analyzed using the International Business Machine Statistical Package for Social Sciences (IBM SPSS) version 22 (SPSS Inc., Chicago, IL, USA). Data were entered, checked for data entry errors, explored and cleaned. The normality of the data was checked using the Kolmogorov-Smirnov test of normality. Descriptive analysis such as frequency, percentage, median, and interquartile range, were used to summarize and explain characteristics of the independent and dependent variables. Chi-square or Fisher’s exact test was conducted to determine the association between the variables.

All tests of significance were based on *p* < 0.05 level and confidence interval of 95% was applied. Variables with a *p*-value of less than 0.25 from the bivariate analysis were subjected to binary logistic regression. Multivariate binary logistic regression was used to determine the predictors of immunization defaulters. Fifteen variables were analyzed using backward and forward likelihood ratio (LR) stepwise selection methods. Both the forward LR and backward LR selected 6 similar significant variables. The 6 significant variables were then analyzed using the ‘Enter’ method to obtain the final model. There was no multicollinearity; and there was no significant interaction between the variables. The final model includes the predictors that influence the immunization defaulters. Adjusted odds ratios (aOR) with 95% confidence intervals (CI) were calculated. *P* values less than 0.05 were considered statistically significant [19].

## Results

### Response rate

**Fig. 1:**
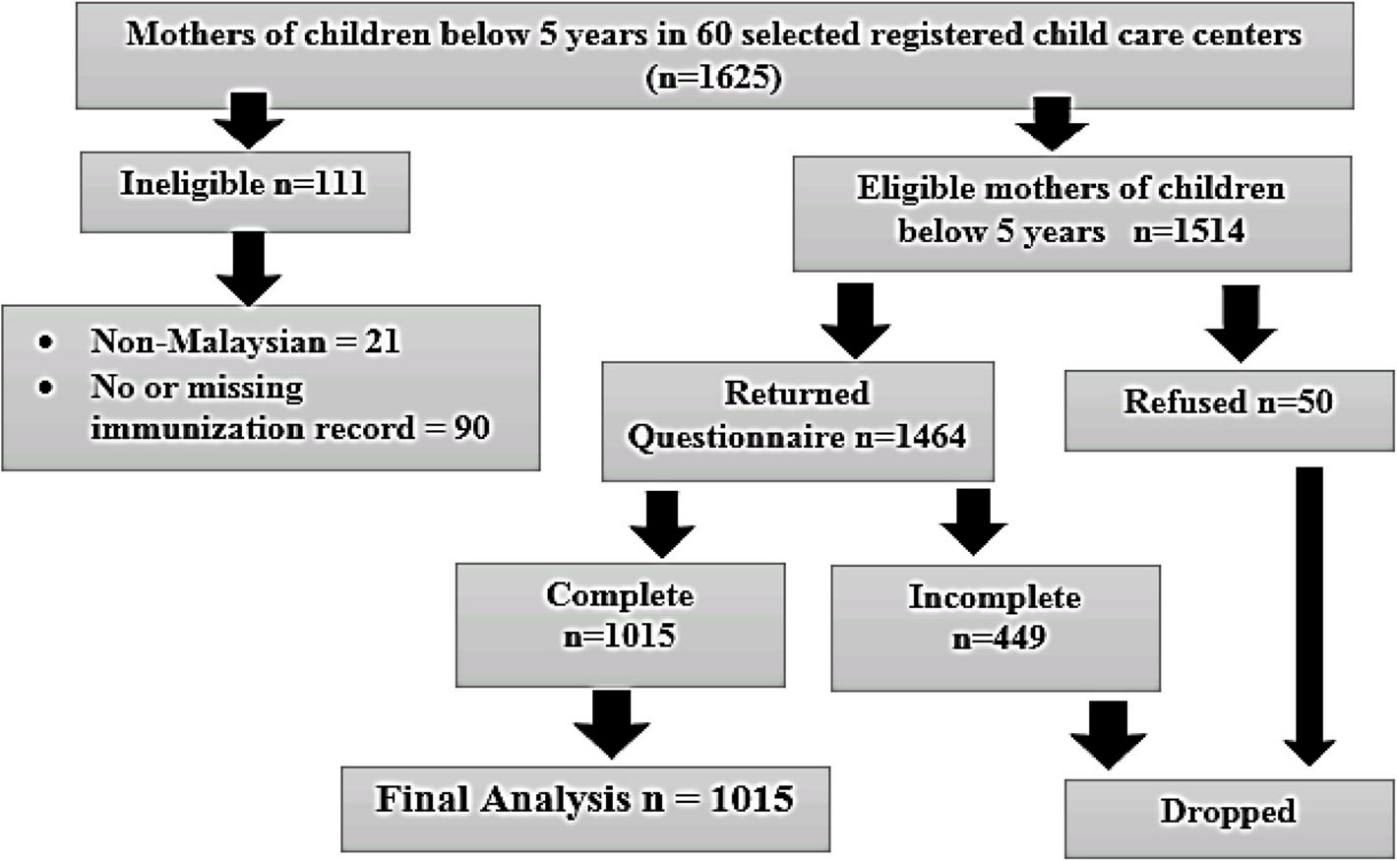
Flow chart of the recruitment of the respondents

Out of 1514 respondents who were selected, 1015 participated and completed the questionnaire in this study, giving an overall response rate of 67%. The remaining (33%) 499 mothers were excluded because the questionnaires returned were incompletely answered (Fig. 1).

### Characteristics of respondents

Table 1 shows the distribution of the respondents by socio-demographic characteristics of mothers and child. Majority of the mothers were between the ages of 30 to 39 years (83%) with median age of 34 years. Most of the respondents were Malay (68.3%) and Muslim (69.7%). A large proportion of the respondents were married, constituting 98.4% of the total respondents whereas the remaining 1.6% was single, divorced or widowed. More than half of the respondents (89.4) had attained tertiary level education of bachelor’s degree and above. In this study, majority of the mothers, 88.9%, were working while the rest, 11.1%, were not working. For family composition, 88.1% of respondents belonged to a family of 5 and below members. Most respondents (36.9%) reported had 2 children. For child factors, a large proportion of the children (79.5) were 2 years or above. The median age for the children was 40 months (IQR: 23). Besides that, nearly half of the children, 494 (48.7%) were male. The highest percentage of the participating children (36.9%) was in the birth order of second.

**Table 1:** Distribution of respondents by socio-demographic characteristic, child factors and healthcare factors (*N* = 1015)

	Frequency (n)	Percentage (%)
Socio-demographic characteristics		
Mother’s age (years)		
20 to 29	107	10.5
30 to 39	842	83.0
40 and above	66	6.5
Ethnicity		
Malay	693	68.3
Chinese	188	18.5
Indian	119	11.7
Others^a^	15	1.5
Religion		
Muslim	707	69.7
Buddhist	160	15.8
Hindu	100	9.9
Christian	45	4.5
Others^b^	3	0.3
Marital Status		
Married	999	98.4
Single/Divorced/Widowed	16	1.6
Educational Level		
Secondary school	28	2.8
Pre-university	13	1.3
Certificate or Diploma	67	6.6
Bachelor’s Degree	696	68.6
Postgraduate studies	211	20.8
Working Status		
Working	902	88.9
Not-working	113	11.1
Family Composition		
5 and below	894	88.1
Above 5	121	11.9
Number of Children		
1	359	35.4
2	375	36.9
3 and above	281	27.7
Child Factors		
Child’s Age		
2 years and below	208	20.5
Above 2 years	807	79.5
Child’s Gender		
Male	494	48.7
Female	521	51.3
Child’s Birth Order		
First	359	35.4
Second	375	36.9
Third and above	281	27.7
Healthcare Factors		
Place of Immunization		
Government clinic	639	63.0
Government hospital	77	7.6
Private clinic	330	32.5
Private hospital	148	14.6
Distance to health facility (km)		
1 to 5	580	57.1
6 to 10	253	24.9
More than 10	182	17.9
Travelling time (minutes)		
Less than 15	461	45.4
15 to 29	378	37.2
30 and above	176	17.3
Waiting time (minutes)		
Less than 60	348	34.3
60 to 119	329	32.4
120 and above	338	33.3
Delayed immunization schedule		
Yes	210	20.7
No	805	79.3

^a^Other ethnicities were Orang Asli, Dusun, Rungus, Kadazan, Iban and Punjabi

^b^Other religions were no religion, Sikh and Bahai

The distribution of respondents according to place of immunization indicated that more than half of the respondents, 63%, immunize their children mainly in government clinic. As for the distance to the preferred immunization health facility, 57.1% of the participating mothers reported a distance of 1 to 5 km (57.1%) with a median distance of 5 km (IQR 7 km). Nearly half of the total respondents travelled less than 15 min to the regularly visited immunization health facility (45.4%). The median travelling time was 15 min (IQR 10). Particularly, 34.1% of the mothers reported that they waited less than 60 min with a median time of 60 min (IQR 90). Amongst all the 1015 children, 20.7% of them have delayed their immunization schedule by over 30 days for at least one dose of immunization and the rest had received all the recommended immunizations within 1 month of due date (79.3%).

### Prevalence of childhood immunization

The prevalence of childhood immunization among children aged 5 years and below attending child care centers in Petaling District was 20.7% for defaulters and 79.3% for non-defaulters. From the 210 respondents in the defaulter group, DTaP/IPV/Hib booster dose was most frequently defaulted in 131 (62.4%) children, followed by DTaP/IPV/Hib third dose in 124 (59%) children. Among the doses of Hepatitis B immunization, the third dose had the most defaulters (22.4%) followed by the second dose (10.5%) while the least rate of defaulters was for the first dose (4.8%). The rate of defaulters for MMR immunization was 24.3%. In addition, 15 children had not received BCG immunization against tuberculosis (Table 2).

**Table 2:** Distribution of defaulters according to types of immunization (*n* = 210)

Type of Immunization	Recommended age given (months)	n	Percentage of defaulting %
BCG	Birth	15	7.1
Hepatitis B – 1st dose	Birth	10	4.8
Hepatitis B – 2nd dose	1	22	10.5
Hepatitis B – 3rd dose	6	47	22.4
DTaP/IPV/Hib 1st dose	2	84	40.0
DTaP/IPV/Hib 2nd dose	3	100	47.6
DTaP/IPV/Hib 3rd dose	5	124	59.0
MMR	12	51	24.3
DTaP/IPV/Hib booster dose	18	131	62.4

*BCG* Bacillus Calmette–Guérin, *HepB* Hepatitis B, *DTaP* Diphtheria, Tetanus, Acellular Pertussis, *Hib Haemophilus influenzae* b, *IPV* Inactivated Poliovirus, *MMR* Mumps, Measles, Rubella

Table 3 shows the factors associated with immunization defaulters. The results show that there was a significant association between immunization defaulters and maternal age (χ2 = 18.811, *df* = 2, *p* < 0.0001), ethnicity (χ2 = 22.325, *df* = 1, *p* < 0.0001) and religion (χ2 = 14.096, *df* = 1, *p* < 0.0001). According to age group, the mothers in the older group tend to have higher percentage of defaulters. Between ethnicity and religion, there were more defaulters among the Non-Malay and Non-Muslim group respectively. Among mothers living without partners such as those who were singled, divorced or widowed, 81.3% of them were found in the defaulter group, whereas only 18.8% of them were found in the non-defaulter group and this difference was statistically significant (Fisher Test *p* < 0.0001).

**Table 3:** Factors associated with immunization defaulters (*N* = 1015)

Socio-demographic characteristics	Defaulter n (%)	NonDefaulter n (%)	*p* value
Mother’s age (years)			
20 to 29	25 (23.4)	82 (76.6)	< 0.0001*
30 to 39	158 (18.8)	684 (81.2)	
40 and above	27 (40.9)	39 (59.1)	
Ethnicity			
Malay	115 (16.6)	578 (83.4)	< 0.0001*
Non-Malay	95 (29.5)	227 (70.5)	
Religion			
Muslim	124 (17.5)	583 (82.5)	< 0.0001*
Non-Muslim	86 (27.9)	222 (72.1)	
Marital status			
Married	197 (19.7)	802 (80.3)	< 0.0001*^+^
Single/divorcee/widow	13 (81.3)	3 (18.8)	
Educational level			
Diploma and below	47 (43.5)	61 (56.5)	< 0.0001*
Degree and above	163 (18.0)	744 (82.0)	
Working status			
Working	175 (19.4)	727 (80.6)	0.004*
Not-working	35 (31.0)	78 (69.9)	
Family composition			
5 and below	168 (18.8)	726 (81.2)	< 0.0001*
Above 5	42 (34.7)	79 (65.3)	
Number of children			
1 to 2	129 (17.6)	605 (82.4)	< 0.0001*
3 to 4	72 (27.6)	189 (72.4)	
5 or higher	9 (45.0)	11 (55.0)	
Child’s age			
2 years and below	56 (26.9)	152 (73.1)	0.013*
Above 2 years	154 (19.1)	653 (80.9)	
Child’s gender			
Male	116 (23.5)	378 (76.5)	0.032*
Female	94 (18.0)	427 (82.0)	
Child’s birth order			
First born	52 (14.5)	307 (85.5)	< 0.0001*
Non-first born	158 (24.1)	498 (75.9)	
Place of immunization			
Government	102 (17.9)	468 (82.1)	0.042*
Private	84 (24.7)	256 (75.3)	
Mixed	24 (22.9)	81 (77.1)	
Distance to health facility (km)			
1 to 5	98 (16.9)	482 (83.1)	0.001*
More than 5	112 (25.7)	323 (74.3)	
Travelling time to health facility (minutes)			
Less than 15	63 (13.7)	398 (86.3)	< 0.0001*
15 to 29	96 (25.4)	282 (74.6)	
30 and above	51 (29.0)	125 (71.0)	
Waiting time (minutes)			
Less than 60	83 (23.9)	265 (76.1)	0.199
60–119	63 (19.1)	266 (80.9)	
120 and above	64 (18.9)	274 (81.1)	
Delayed immunization schedule			
Yes	84 (40.0)	126 (60.0)	< 0.0001*
No	126 (15.7)	679 (84.3)	

*Significant at *p* value < 0.05

Maternal highest educational level was also found to have a statistically significant association with immunization defaulters (χ2 = 38.386, *df* = 1, *p* < 0.0001). Specifically, 43.5% of mothers with diploma and below educational background had defaulted on their children’s immunization compared to 18.0% of mothers who completed at least bachelor’s degree or postgraduate studies. Mother’s working status also showed a significant difference between the two groups in which 31.0% of mothers who were not working defaulted compared to 19.4% of working mothers defaulted (χ2 = 8.195, *df* = 1, *p* = 0.004). The difference between immunization defaulters of children belonging to a larger family size of more than 5 members and smaller family size with at least 5 members or less found to be significant (χ2 = 16.459, *df* = 1, *p* < 0.0001). A significant association was found between the number of children within the family with immunization defaulters (χ2 = 19.108, *df* = 2, *p* < 0.0001). The more number of children within a family, the higher the percentage of immunization defaulters.

Besides, the result also shows that there was a significant association between childhood immunization defaulters and child’s age (χ2 = 6.195, df = 1, *p* = 0.013), gender (χ2 = 4.572, *df* = 1, *p* = 0.032) and birth order (χ2 = 13.033, *df* = 1, *p* < 0.0001). The result also shows that place of immunization (χ2 = 6.356, *df* = 2, *p* = 0.042), distance (χ2 = 11.866, *df* = 1, *p* = 0.001), travelling time (χ2 = 26.331, *df* = 2, *p* < 0.0001) and delayed immunization schedule (χ2 = 60.171, *df* = 1, *p* < 0.0001) were found to have significant association with immunization defaulters. However, there was no significant association observed between the waiting time to get the child immunized and childhood immunization (*p* > 0.05).

In the binary regression logistic analysis, independent variables that were possibly associated with childhood immunization defaulters were entered into the logistic regression model. There was no multicollinearity; and there was no significant interaction between the variables. The variables that were retained in the final logistic regression model were religion, educational level, number of children, child’s age group, travelling time to health facility and delayed immunization schedule.

Table 4 presents the results of binary logistic regression to determine the predictors of childhood immunization defaulters. The predictors of childhood immunization defaulters were non-Muslims (adjusted OR = 1.669, 95% CI = 1.173, 2.377, *p* = 0.004), mothers with diploma and below educational background (adjusted OR = 2.296, 95% CI = 1.460, 3.610, *p* < 0.0001), multiple children of 5 and above in a family (adjusted OR = 2.656, 95% CI = 1.004, 7.029, *p* = 0.040), mothers with younger children aged 2 years and below (adjusted OR = 1.700, 95% CI = 1.163, 2.486, *p* = 0.006), long travelling time of more than 30 min to the immunization health facility (adjusted OR = 2.303, 95% CI = 1.474, 3.599, *p* < 0.0001) and delayed at least one of the immunization schedule (adjusted OR = 2.747, 95% CI = 1.918, 3.933, *p* < 0.0001).

**Table 4:** Binary logistic regression analysis of predictors of immunization defaulters

Factors	Adjusted OR	95% CI	*p* value
Religion			
Muslim	1		
Non-Muslim	1.669	1.173–2.377	0.004*
Educational Level			
Diploma and below	2.296	1.460–3.610	0.011*
Bachelor’s degree and above	1		
Number of Children			
1 to 2	1		
3 to 4	1.599	1.109–2.307	0.012*
5 or higher	2.656	1.004–7.029	0.040*
Child’s Age			
2 years and below	1.700	1.163–2.486	0.006*
Above 2 years	1		
Travelling time to health facility (minutes)			
Less than 15	1		
15–29	1.811	1.247–2.628	0.002*
30 and above	2.303	1.474–3.599	< 0.0001*
Delayed immunization schedule			
Yes	2.747	1.918–3.933	< 0.0001*
No	1		

*OR* Odds ratio, *CI* Confidence interval, 1: reference group, *Significant at *p* value < 0.05

The significance levels of the Wald statistics demonstrated that all the 6 predictor variables made a significant contribution to immunization defaulters after adjusting other variables. The Hosmer-Lemeshow test showed a *p*-value of 0.421 which means that it is not statistically significant, implying that the logistic regression model was statistically significant and fits the sample (χ2 = 7.078, *df* = 7, *p* = 0.421). This study found that those factors can explain 16.6% of the variation in the factors influencing immunization defaulters.

## Discussion

Based on previous Malaysian studies, prevalence of childhood immunization defaulters ranges from 16.8 to 24.8% [11, 18]. The results of this study identified that the prevalence of immunization defaulters among children below 5 years attending registered child care centers in Petaling District, Selangor was 20.7%, falling within that range. This shows that 20.7% of the children in this study had default or missed at least one of the recommended immunization schedules, thus making them unprotected or inadequately protected against these vaccine-preventable diseases.

The proportion of children who had defaulted immunization varied for the different vaccines doses. The most commonly defaulted immunization in defaulters group is DTaP/IPV/Hib booster dose. This shows that the majority of the defaulted children were lack of booster dose to fully protect them against a vaccine preventable disease. The lack of awareness on the necessity to return for booster vaccine doses may influence mothers not to bring their children again for immunization. In this study, a consistent increase in immunization defaulters from the first to the third dose was observed in DTaP/IPV/Hib (40.0, 47.6, 59.0%) and Hepatitis B (4.8, 10.5, 22.4%) vaccine, respectively. This shows that a repeated dose of an immunization increased the rate of defaulting subsequent doses, similar to what has been reported by Azhar et al. among children in Sabah, Malaysia [11]. The possible explanation for such finding could be due to the result of time gap between two vaccine leading mothers to forget the subsequent dose [11].

Waiting time was not a significant factor for childhood immunization defaulters. The possible explanation for the insignificant result in this study could be due to the availability of the immunization health facilities approximately within five kilometers from their houses and free immunization provided by government hospitals or clinics to the children may have benefited the mothers and outweighed the risks of defaulting immunization. The result contradicts with the results found by Lim et al. [20] and Abdulraheem et al. [21] where the authors found a significant association between waiting time and incomplete immunizations. The differences in the findings could be due to the different study settings by Lim et al. [20] and Abdulraheem et al. [21] as both were conducted in government healthcare centers whereas this study was conducted in child care centers, whereby services were received from government, private or both facilities.

This study pointed out that religion (*p* < 0.0001) was found to be significantly associated with immunization defaulters. Similar findings were also observed by other researchers in Australia and India [22–24]. The immunization defaulters were significantly higher among non-Muslim respondents compared to respondents of Muslim religious belief from the majority ethnic group. This finding was similar and supported by a recent Malaysian study on vaccine hesitancy among parents in a tertiary hospital in Kuala Lumpur which found that non-Muslim parents were significantly more vaccine hesitant than Muslim parents (*p* < 0.026) [25]. The possible reason could be due to the vaccine safety concern that may influence the immunization decision among the non-Muslims primarily Chinese and Indian parents [26].

Further, the results found a significant association between maternal education levels with immunization defaulters in children. The importance of education level in influencing immunization status among children was also proven by studies from other developing countries [27–30]. This study shows that children borne from less educated mothers, with diploma and below, tend to default immunization schedule compared to children borne from more educated counterparts, with bachelor’s degree and above. Mother’s educational level could also influence the knowledge one has on childhood immunization which could eventually influence one’s decision on their children’s health [31]. Maternal education influences child health-care-seeking behavior whereby education increases knowledge and enables better understanding on immunization among mothers with higher education level [30].

This study revealed that the number of children was significantly associated with immunization defaulters. It was found that odds of a child being a defaulter increases with the number of children in the household. Similar findings were obtained from other studies in Malaysia that shows that multiple children were a contributing factor to incomplete immunization [18, 25]. This could suggest that greater number of children in families placed heavier burden on mothers, hence reducing the quality of care received by the children [32]. Besides that, this study also revealed that there is a significant association between child’s age and immunization defaulters. Younger children below 2 years were found to have an increased likelihood of being immunization defaulters. This study is in accordance with other studies that shows child’s age as a significant factor for defaulting immunization [18, 33]. The reason could be due to the recent huge amounts of conflicting vaccine-safety information and misinformation on the Internet that could negatively influence current mothers’ decision to immunize their children [34].

The results of this study are consistent with other recent studies in developing countries from Ethiopia [35] and Cameroon [36] which have also suggested that traveling time to the immunization sites had significant association with the utilization of immunization services. In the multivariate results, immunization defaulters increased with increasing traveling time to the immunization health facility. This could be due to the fact that if a health facility is located within a convenient proximity, it is likely to motivate mothers to adhere to the immunization schedule [35]. The findings from this study also showed that there was a significant association between delayed immunization schedule and immunization defaulters. A similar study in Ethiopia showed that delaying child immunization schedule was a predictor of defaulting from completion of child immunization [37]. Children whose mothers delay immunization may be at an increased risk of not receiving all recommended vaccine doses and may leave their children more vulnerable to vaccine-preventable diseases [38, 39], suggesting that mothers delayed immunization because of concerns about vaccine safety and child illnesses [38]. Thus, it is important that a child receives a complete series of immunizations according to the schedule.

### Strengths and limitations

There are some limitations in this study that warrant discussion. Firstly, its cross-sectional study design does not allow causality to be established. Secondly, data collection was confined to registered child care centers in one district of Malaysia. Therefore, the study population may not represent the whole country. However, the results from this study could be said to represent mothers at-risk in Malaysia. As immunization status was assessed using only the child’s immunization record, mothers without their child’s immunization records were not included in this study and could have led to information biasness. There is a possibility that this group might not have received immunization as scheduled and thus escaped the analysis for identification of immunization defaulters. However, studies show that mothers’ responses are accurate and provide generally adequate information even if they are said to underreport immunization uptakes [40, 41].

### Recommendations

The following recommendations are suggested; as this was a cross-sectional study with inherent study limitations, prospective studies should be carried out with the focus on modifiable and non-modifiable risk factors for immunization defaulters among young children to strengthen the findings of this study. A qualitative study should also be considered to explore in depth on mothers’ perception, acceptance, barriers and values placed on childhood immunization. Based on the finding of the predictors of immunization defaulters in this study, it is recommended to develop short community based educational programs to educate mothers, especially those in the high-risk group such as Non-Muslims, lower educational levels, multiple children and with younger children. The program should also include the risk of delaying immunization schedule and the benefits of completing the administration of immunization for children. Moreover, the mothers in the high-risk group should be given special attention and encouragement to immunize their children. An effective vaccine reminder system could be implemented, in order to encourage appropriate administration of booster doses to educate mothers at risk of defaulting immunization.

## Conclusion

This study shows that monitoring the immunization status among children is important and necessary, as 20.7% of them were defaulters. The modifiable predictors of immunization defaulters in this study were traveling time to health facility, delayed immunization schedule while religion, educational level, number of children and child’s age were the non-modifiable predictors of immunization defaulters in this study.

Follow-up systems, counseling on childhood immunization, and effective intervention programs designed to improve childhood immunization uptake among mothers are also needed, especially among non-Muslims, mothers with educational level of diploma and below, mothers with more than 5 children and mothers with children aged 2 years and below. Identifying mothers at risk of not completing their children’s immunization schedule and educating them is an important strategy to prevent outbreaks of infectious diseases in the country.

## Additional files

Additional file 1: *Questionnaire, *English language version. (PDF 267 kb)

## Data Availability

The datasets used and/or analyzed during the current study are available from the corresponding author on reasonable request.
