# Supplementary material for: Sociodemographic and health care factors in determining immunization defaulters among preschool children in Petaling District, Selangor: a cross-sectional study in Malaysia
Source: BMC Public Health. 2019 Sep 18;19:1275. doi: 10.1186/s12889-019-7561-z (PMC6751745; doi:10.1186/s12889-019-7561-z)
Supplement: Supplementary file 1 — *Questionnaire, *English language version. (PDF 267 kb) [file 12889_2019_7561_MOESM1_ESM.pdf]

Part A: Personal Background Details

This section is about you and **your youngest or last born child.**
Please mark (√) in the appropriate box and write your answer in the space provided.

1. Mother's Age, in [date/month/year] : ……….. /…………. /………….

2. Ethnicity :

   - ❑ Malay
   - ❑ Chinese
   - ❑ Indian
   - ❑ Others (please specify) : ………………………..

3. Religion :

   - ❑ Islam
   - ❑ Buddha
   - ❑ Hindu
   - ❑ Christian
   - ❑ Others (please specify) : ………………………..

4. Marital Status :

   - ❑ Single
   - ❑ Married
   - ❑ Divorcee
   - ❑ Widow

5. Highest Educational Level :

   - ❑ Primary school
   - ❑ Secondary school
   - ❑ Pre-University (contoh / example: STPM, A-Level)
   - ❑ Certificate or Diploma
   - ❑ Bachelor Degree
   - ❑ Postgraduate studies (contoh / example: Masters, PhD)

6. Employment status :

- ❑ Employed
- ❑ Self-employed
- ❑ Unemployed

7. Family members : …………………members

8. Number of children, excluding current pregnancy (if any): ……………children

9. Youngest or last born child's birth date,
   in [date/month/year]: ……….. /…………. /………….

10. Youngest or last born child's gender :

- ❑ Male
- ❑ Female

11. Child's birth order : …………………order

Part B: Your Child's Immunization History

Please mark (√) in the appropriate box and write your answer in the space provided.

12. Have you ever immunize your youngest child?
    - ❑ Yes (Please proceed to Question 12)
    - ❑ No (Please proceed to Question 13)

13. Please tick (√) in the appropriate boxes below, for all the type of immunization that your YOUNGEST OR LAST BORN CHILD has received till now. (Use your child's immunization record as reference).

Type of immunization	Taken	Didn't Take	If Taken	
			Date of immunization given by clinic staff	Date of immunization taken by child
BCG				
Hepatitis B (Dose1)				
Hepatitis B (Dose 2)				
Hepatitis B (Dose 3)				
DTaP/DT (Dose 1)				
DTaP/DT (Dose 2)				
DTaP/DT (Dose 3)				
DTaP/DT (Booster)				
Hib (Dose 1)				
Hib (Dose 2)				
Hib (Dose 3)				
Hib (Booster)				
IPV/OPV/Polio (Dose 1)				
IPV/OPV/Polio (Dose 2)				
IPV/OPV/Polio (Dose 3)				
IPV/OPV/Polio(Booster)				
MMR (Dose 1)				

Part C: Immunization Health Facility

This section is only for those with child who taken at least one immunization.
Please mark (√) in the appropriate box and write your answer in the space provided.

15. Place of immunization regularly visited for immunization of your child:
    (You are allowed to answer more than 1 choice of answer)
    - ❑ Government clinic
    - ❑ Government hospital
    - ❑ Private clinic
    - ❑ Private hospital

16. Estimated distance to the regularly visited immunization clinic or hospital to get immunization for your child : …………………....kilometer (km)

17. Estimated travelling time taken from home to reach the regularly visited immunization clinic or hospital to get immunization for your child : ……………………….minutes

18. Estimated waiting time spent to get your child immunized at the regularly visited immunization clinic or hospital to get immunization for your child : ..…………………minutes

The End
